# Intrinsic Immunogenic Tumor Cell Death Subtypes Delineate Prognosis and Responsiveness to Immunotherapy in Lung Adenocarcinoma

**DOI:** 10.3390/biology12060808

**Published:** 2023-06-02

**Authors:** Xiaotian He, Dechang Zhao, Xuewen Zhang, Yiyang Ma, Rusi Zhang, Zirui Huang, Gongming Wang, Guangran Guo, Weidong Wang, Yingsheng Wen, Lanjun Zhang

**Affiliations:** 1 State Key Laboratory of Oncology in South China, Collaborative Innovation Center for Cancer Medicine, Guangzhou 510060, China; hext@sysucc.org.cn (X.H.); zhaodc@sysucc.org.cn (D.Z.); zhangxuew@sysucc.org.cn (X.Z.); mayy1@sysucc.org.cn (Y.M.); zhangrs@sysucc.org.cn (R.Z.); huangzr@sysucc.org.cn (Z.H.); wanggm@sysucc.org.cn (G.W.); guogr@sysucc.org.cn (G.G.); wangweid@sysucc.org.cn (W.W.); 2 Department of Thoracic Surgery, Sun Yat-sen University Cancer Center, Guangzhou 510060, China; 3 Department of Anesthesiology, Sun Yat-sen University Cancer Center, Guangzhou 510060, China

**Keywords:** immunogenic cell death, immunotherapy, lung adenocarcinoma, prognosis, tumor microenvironment

## Abstract

**Simple Summary:**

Intrinsic immunogenic cell death (ICD) property plays an important role in the prognosis and immune microenvironment of patients with lung adenocarcinoma (LUAD). In this study, we performed an overall multi-omics analysis of the intrinsic ICD property and developed a risk scoring system. We found two distinct ICD-associated transcriptomic molecular patterns (termed ICD-high and ICD-low). We identified and validated ICDrisk subtypes (ICDrisk) which can effectively predict overall survival (OS) in LUAD patients and immunotherapeutic response across Pan-cancer. Our results may help to elucidate the underlying molecular mechanisms of intrinsic immunogenicity and heterogeneous responses to immunotherapy in LUAD patients.

**Abstract:**

Recent studies have highlighted the combination of activation of host immunogenic cell death (ICD) and tumor-directed cytotoxic strategies. However, overall multiomic analysis of the intrinsic ICD property in lung adenocarcinoma (LUAD) has not been performed. Therefore, the aim of this study was to develop an ICD-based risk scoring system to predict overall survival (OS) and immunotherapeutic efficacy in patients. In our study, both weighted gene co-expression network analysis (WGCNA) and LASSO-Cox analysis were utilized to identify ICDrisk subtypes (ICDrisk). Moreover, we identify genomic alterations and differences in biological processes, analyze the immune microenvironment, and predict the response to immunotherapy in patients with pan-cancer. Importantly, immunogenicity subgroup typing was performed based on the immune score (IS) and microenvironmental tumor neoantigens (meTNAs). Our results demonstrate that ICDrisk subtypes were identified based on 16 genes. Furthermore, high ICDrisk was proved to be a poor prognostic factor in LUAD patients and indicated poor efficacy of immune checkpoint inhibitor (ICI) treatment in patients with pan-cancer. The two ICDrisk subtypes displayed distinct clinicopathologic features, tumor-infiltrating immune cell patterns, and biological processes. The IS^low^meTNA^high^ subtype showed low intratumoral heterogeneity (ITH) and immune-activated phenotypes and correlated with better survival than the other subtypes within the high ICDrisk group. This study suggests effective biomarkers for the prediction of OS in LUAD patients and immunotherapeutic response across Pan-cancer and contributes to enhancing our understanding of intrinsic immunogenic tumor cell death.

## 1. Introduction

Lung cancer is the leading cause of cancer-related death and the second most commonly diagnosed cancer [1]. Among all lung cancer subtypes, lung adenocarcinoma (LUAD) is the most common subtype with a poor prognosis and high recurrence and metastasis rates [2]. Therefore, improving the survival rate of LUAD patients remains a great challenge.

Due to the wide variation in intratumor heterogeneity impacting the prognosis of LUAD patients, it is difficult to accurately assess the prognosis of patients only with the AJCC TNM staging system [3]. Therefore, it has become necessary to incorporate other important factors to achieve accurate and personalized evaluations. In recent decades, many emerging therapies have effectively improved the survival of LUAD patients, including molecular targeted therapy and immune checkpoint inhibitors (ICIs) [4]. At present, many biomarkers have been indicated to be effective in evaluating the efficacy of ICI treatments, including programmed death-ligand 1 (PD-L1) and the tumor mutational burden (TMB) [5]. However, many studies recently have confirmed the deficiencies of PD-L1 and the TMB in predicting response to immunotherapy in LUAD patients, which may cause LUAD patients to lose the opportunity to be treated with immunotherapy [6,7]. Therefore, it is necessary to explore more effective biomarkers to predict the response to ICIs in LUAD patients.

ICIs therapy was initially found to promote CD8+ T-cell activity and alleviate the depletion of antitumor T cells, so it seems reasonable to evaluate ICB therapy based on CD8+ T lymphocyte infiltration and function [8]. However, due to the complex interactions between immune cells and tumor cells, the effect of immune checkpoint blockade (ICB) in the treatment of cancer is highly heterogeneous. There is increasing evidence that tumors with low rates of T-cell infiltration may respond reasonably well to ICB, whereas tumors with high rates of T-cell infiltration may not necessarily respond well to ICB [9,10]. The intrinsic immunogenicity of tumor cells in the tumor microenvironment (TME) is also a crucial factor affecting immunotherapy and even affects the prognosis of patients [11].

Immunogenic cell death (ICD) recently has been proven to be a cell death modality that can drive an adaptive immune response [12]. Briefly, dead cells release damage-associated molecular patterns (DAMPs) and cytokines to drive inflammatory responses, which may eventually activate the cytotoxic T cell (CTL)-driven acquired immune response while establishing long-term immune memory. There is ample evidence that anticancer immune responses caused by ICD inducers can reinforce the curative effect of anticancer therapies, including chemotherapy, radiotherapy, and immunotherapy, in many preclinical models [13,14,15]. However, only a few ICD inducers have been proven to be effective in clinical anticancer therapies, especially in immunotherapy [16]. Both TME-intrinsic factors and TME-extrinsic factors contribute to the response to ICB and the prognosis of patients. At present, few studies have identified intrinsic ICD-associated molecular patterns. Therefore, understanding the intrinsic ICD features in NSCLC is a critical area that remains to be explored.

In our study, we systematically characterized the ICD level based on 34 ICD metagenes, which have been summarized in previous studies. Two distinct ICD-associated transcriptomic molecular patterns (termed ICD-high and ICD-low) in the TCGA-LUAD cohort were identified by WGCNA. Moreover, we established an ICDrisk scoring system to assess the prognostic value in LUAD patients, identify genomic alterations and differences in biological processes, analyze the immune microenvironment, and predict the response to immunotherapy in patients with pan-cancer. Subgroup analysis based on intrinsic immunogenicity found that a low immune score and high neoantigen count subcluster indicated a better prognosis. Our results may help to elucidate the underlying molecular mechanisms of the intrinsic immunogenicity and heterogeneous responses to immunotherapy in LUAD patients, thereby further improving immunotherapy in LUAD patients.

## 2. Materials and Methods

### 2.1. Datasets and Preprocessing

The Cancer Genome Atlas (TCGA) cohort from the “TCGA-Lung Adenocarcinoma Carcinoma” (TCGA-LUAD) project transcriptomic datasets (FPKM format) was used. Genomic Data Commons Data Portal (https://portal.gdc.cancer.gov/, accessed on 1 May 2021) was used to download gene somatic mutations and follow-up data for LUAD patients in November 2021. Several representative Gene Expression Omnibus (GEO) datasets (http://www.ncbi.nlm.nih.gov/geo, accessed on 1 May 2021) that contained large populations of patients with LUAD (n > 80) with clinical information were enrolled as the public validation cohorts, which included 442 cases from GSE68465, 398 cases from GSE72094, and 86 cases from GSE68571. Between September 2012 and December 2017, 78 clinical samples were collected at the Sun Yat-sen University Cancer Center (SYSUCC). Clinical and pathologic information was collected during postoperative follow-up. This study was approved by the Ethics and Research Committees of the Sun Yat-sen University Cancer Center. We selected 11 public independent pan-cancer immunotherapy cohorts for external validation of the risk scoring system, including 3 NSCLC cohorts (GSE93157, GSE126044, and GSE135222), 3 urologic tumor cohorts (IMvigor210, JAVELIN Renal 101, and RCC-Braun_2020), and 5 melanoma cohorts (GSE91061, GSE93157, Melanoma-Nathanson, Melanoma-phs000452, and PRJEB23709 datasets) (Table S1). Information on clonal/subclonal mutations, neoantigen counts, and tumor heterogeneity was obtained from The Cancer Immunome Atlas (TCIA, https://tcia.at/, accessed on 1 May 2021) [17].

### 2.2. Identification of the ICD Level in TCGA-LUAD Samples

A large-scale meta-analysis summarized immunological metagene signatures for immunogenic cell death [18]. We extracted 34 ICD-related parameters as a gene set and single-sample GSEA (ssGSEA) was used to calculate the ICD level for each sample with the R package “GSVA”, this method identifies genes whose expression is coordinated within a sample [19].

### 2.3. WGCNA and Construction of a Scoring System to Assess Single-Sample Immunogenic Cell Death (ICD) Patterns

Co-expressed gene modules closely related to ICD scores were identified using WGCNA. First, we screened whole coding genes from the TCGA-LUAD dataset, calculated the median absolute deviation of each mRNA, sorted by deviation from large to small, and selected the top 75% of the genes for WGCNA which was performed using the WGCNA R package [20]. We first choose a soft threshold of co-expression similarity to compute adjacency. We set the soft threshold to 5, R square = 0.88. After the adjacency matrix was transformed, a topological overlap matrix (TOM) was obtained, which was then used as the input for hierarchical clustering. Then, module eigengenes (MEs) with ≥30 genes were selected using the dynamic tree-cut method. We merged modules with similar expression profiles based on a threshold of 0.25. Gene significance (GS) and module membership (MM) were used to distinguish the vital modules associated with immunogenic cell death. GS > 0.6 and MM > 0.6 were considered to identify strong ICD-related modules. Subsequently, ICD-related genes were chosen from ICD-correlated modules. We used ssGSEA to evaluate the ICD score of individual lung adenocarcinoma patients based on the gene expression levels of ICD-related genes in a single sample, and we identified patients as ICD high (ICD-H) and ICD low (ICD-L).

### 2.4. Functional and Pathway Enrichment Analysis

ICD-related genes were analyzed using the Gene Ontology (GO) and Kyoto Encyclopedia of Genes and Genomes (KEGG) databases [21,22]. The above functional enrichment analysis was performed using the R package ‘clusterProfiler’, and the results were visualized using the R package ‘ggplot2’.

For gene set enrichment analysis (GSEA), we used the GSEA software (version 3.0) on the GSEA website (http://software.broadinstitute.org/gsea/index.jsp, accessed on 1 May 2021) [23]. Potential biological functions were identified using the GSEA method and annotated by Hallmark gene sets (https://www.gsea-msigdb.org/gsea/msigdb/index.jsp, accessed on 1 May 2021) and the GO and KEGG databases.

### 2.5. Detecting Differentially Expressed Genes (DEGs) and Protein-Protein Interaction (PPIs)

Transcriptomic data were imported into R software, and the “limma” package was employed to identify DEGs in different clusters. We constructed a PPI network from DEGs identified between different ICD scores using the STRING interactome (http://string-db.org, accessed on 1 May 2021). Using Cytoscape software with the molecular complex detection (MCODE) plug-in, the core protein-protein interaction network complex of these DEGs was identified.

### 2.6. Establishment and Verification of an ICDrisk Model

Univariate Cox regression analysis was used to identify associations between the expression levels of ICD-related genes in the TCGA-LUAD cohort RNA-seq dataset and patient prognosis. Then, in order to reduce overfitting and eliminate tightly correlated genes, LASSO Cox regression models were used to analyze potential prognostic factors. Using tenfold cross-validation, we selected the minimal penalty term (λ). Then, we established an ICDrisk model for LUAD patients containing 16 hub genes. The ICDrisk formula was constructed as follows:

ICDrisk score = ∑i = 1nCoefi × Xi

Coefi is the coefficient and Xi is the normalized count of each hub gene. The median of the training series was used as the cut-off to stratify patients into high- and low- ICDrisk subtypes. Kaplan–Meier survival differences between high- and low-ICDrisk subtypes were further compared in the TCGA-LUAD training set and the GEO validation sets.

### 2.7. Extraction of RNA and qRT-PCR

For total RNA isolation from tissues, TRIzol reagent (Invitrogen, Carlsbad, CA, USA) was used. For cDNA synthesis, HiScript II (R201-01, Vazyme, China) was used. Then, hub genes and GAPDH were quantified with 2X SYBR Green qPCR Master Mix (K1070, APExBIO, Houston, TX, USA). The primers for qRT-PCR were provided by Synbio Technologies. GAPDH was chosen as the internal reference. In Table S2, the primers used for PCR are shown, including the relative mRNA expression of hub genes normalized to GAPDH.

### 2.8. Human Protein Atlas Database Analysis

The KRT16, S100P, CYP24A1, SERPINB5, and KRT6A protein expression in LUAD tissue was explored in The Human Protein Atlas (HPA) database (https://www.proteinatlas.org/, accessed on 1 May 2021) [24]. Immunohistochemistry (IHC) showed a brown color was determined as positive tissue.

### 2.9. Tumor Mutation Status in the Low- and High-ICDrisk Subtypes

We downloaded information for somatic mutations in TCGA-LUAD samples from the Genomic Data Commons Data Portal (https://portal.gdc.cancer.gov/, accessed on 1 May 2021). Mutated genes (*p* < 0.05) identified between different ICDrisk subtypes and the correlation effect of gene mutations were analyzed with the R package ‘maftools’. A one-sided z-test and two-sided chi-square test were used to evaluate the proportion of mutations, and *p* < 0.05 was considered significant.

### 2.10. Estimation of Immune Infiltration and Functional Enrichment for the ICDrisk

The TIMER, CIBERSORT, MCPcounter, QUANTISEQ, and EPIC algorithms were used to compare the components in the immune microenvironment and activation of immune cells between groups at high- and low-risk according to the ICDrisk [25,26,27,28]. The differences in the immune response identified by the different algorithms were visualized using a heatmap. In addition, the ESTIMATE and xCell algorithms were used to robustly quantify tumor purity and the numerous cell populations in the immune microenvironment based on transcriptomic data for each patient [29,30]. The gene expression of the human leukocyte antigens gene family (HLAs) and immune checkpoint molecules were also retrieved from the published literature [31].

### 2.11. TIDE (Tumor Immune Dysfunction and Exclusion) Analysis

The TIDE algorithm is a computational framework to predict cancer immunotherapy response (http://tide. Dfci.harvard.edu, accessed on 1 May 2021). ICDrisk was tested using the TIDE algorithm for predicting clinical responsiveness to ICIs. TIDE can also find the level of T-cell dysfunction and T-cell exclusion which are two primary mechanisms of tumor immune evasion and predicted multiple immunosuppressive cell subgroups, including cancer-associated fibroblasts (CAFs), tumor-associated macrophages (TAMs), and myeloid-derived suppressor cells (MDSCs) [32].

### 2.12. Statistical Analysis

Statistical calculations were performed using R (version 4.1.1, http://www.R-project.org, accessed on 1 May 2021). Patients’ overall survival was evaluated using Kaplan-Meier analysis. Pearson or Spearman correlation analysis was used to generate correlation matrices. We used the Wilcoxon test and the Kruskal-Wallis test to compare continuous and ordered categorical variables, respectively. To adjust the *p*-value for multiple tests, the false discovery rate (FDR) was corrected. *p* < 0.05 was considered statistically significant.

## 3. Results

### 3.1. Calculation of the ICD Level, Performance of WGCNA, and Identification of Key Modules

**Figure 1:**
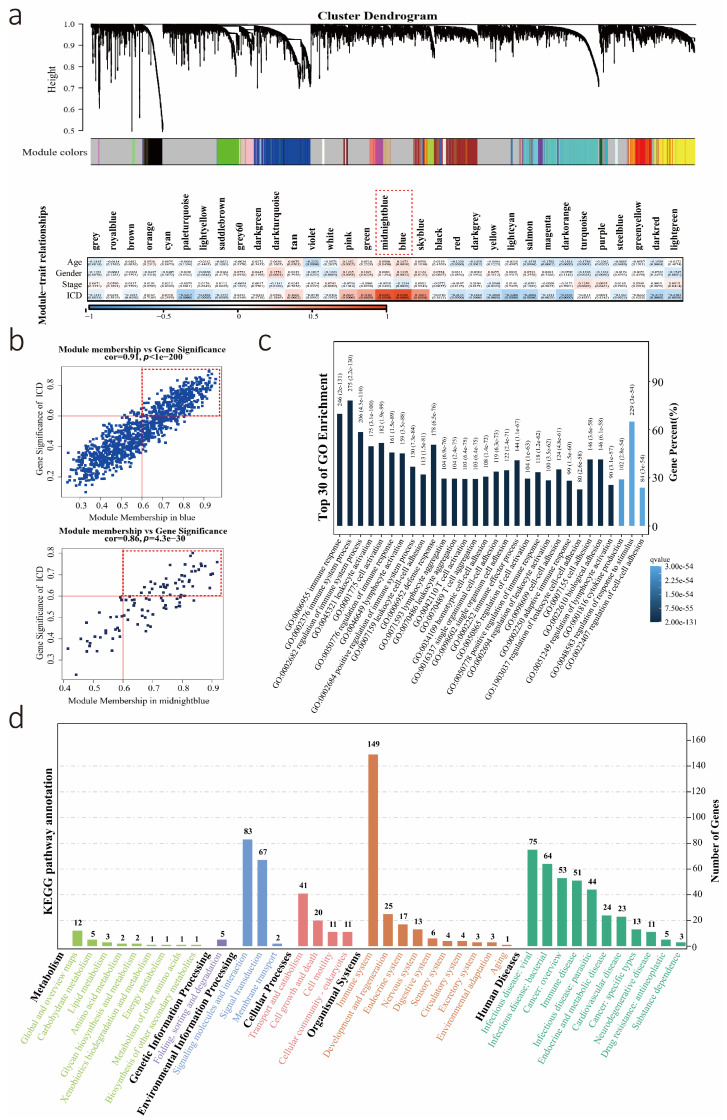
Screening for ICD-related genes. (**a**) Thirty-three co-expressed gene modules identified by WGCNA and Correlation between gene modules and ICD scores. Midnight blue and blue modules are strongly correlated modules (|Cor| > 0.8 and *p* < 0.001) and are marked with red frames. (**b**) Scatted plot represented the relationship between Module Membership (MM) and Gene Significance (GS) in blue and midnight blue. Strongly ICD-related genes were marked with red frames (MM > 0.6 and GS > 0.6). (**c**,**d**) GO and KEGG enrichment analyses were performed to identify the roles of these ICD-related genes in biologically relevant functions.

A total of 510 LUAD patients were collected from the TCGA database in our study. Thirty-four immunological metagene signatures related to immunogenic cancer cell death were used to calculate ICD levels based on the ssGSEA algorithm. The ICD levels of these patients are listed in Table S3. To identify ICD-related modules, the relationship between modules and clinical data was studied. Five was selected as the optimal soft threshold for WGCNA (Figure S1). Thirty-three co-expressed gene modules with *p* < 0.05 were identified, and the midnight blue and blue modules were strongly positively related to the ICD level (Figure 1a). Thus, these two modules were selected as ICD-related modules for further analysis. There was a significant correlation between module membership (MM) and gene significance (GS) in midnight blue and blue modules (Figure 1b). ICD-related genes were screened out with the thresholds GS > 0.6 and MM > 0.6, and 381 ICD-related genes were found in both two modules. Subsequently, we analyzed the roles of these genes in biologically relevant functions. These genes were enriched in multiple important immune-related pathways (Figure 1c,d and Table S4).

### 3.2. Potential Biological Role of the ICD Score as a Predictor

**Figure 2:**
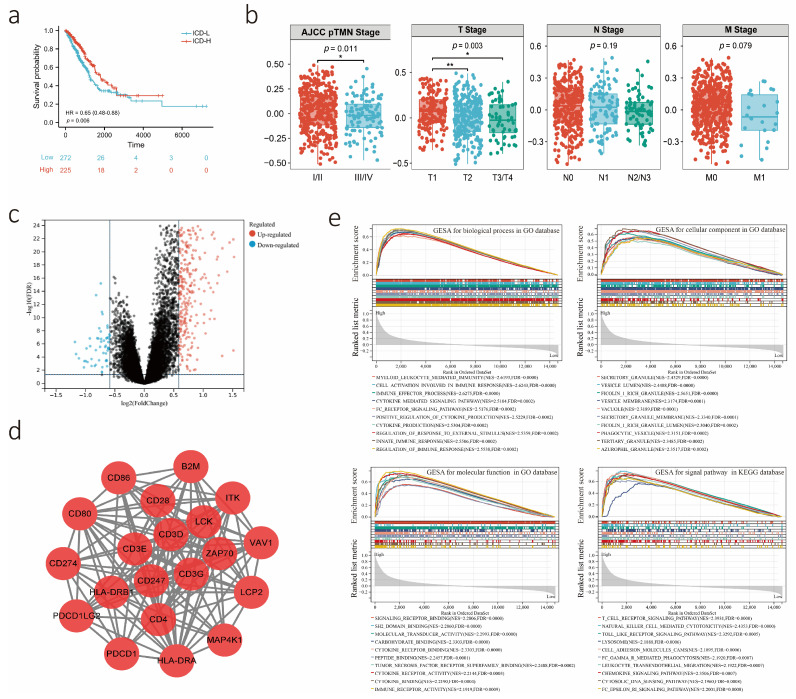
The potential biological role of the ICD score. (**a**) Kaplan-Meier curves showed the OS in ICD-high and ICD-low subgroups. The Red and blue curves represented the ICD-high and ICD-low subgroups respectively. (**b**) The box plot presented the distribution of ICD scores at different stages. (**c**) Compared with the ICD-low subgroup, the volcano plot showed the differentially expressed genes with a threshold of |log2 Fold change| > 0.5 and FDR < 0.05 in the ICD-high subgroup. (**d**) Protein–protein interactions plot showed the hub-gene among ICD-related differentially expressed genes. (**e**) GSEA determined the underlying biological process, cellular component, molecular function, and signal pathway between ICD-high and ICD-low subtypes (|normalized enriched score (NES) | > 1 and FDR value < 0.05). *: *p* < 0.05; **: *p* < 0.01.

We first investigated the relationship between 381 ICD-related genes and the prognosis of patients with lung adenocarcinoma. Owing to the individual variability and complexity of the prognosis of LUAD patients, we construct a scoring system using ssGSEA and quantified the prognosis of each sample, which we called the ICD score. Survival analysis by ICD score demonstrated that the group with low ICD score had poor prognostic survival in the TCGA-LUAD cohort (*p* = 0.006, HR = 0.65, 95% CI: 0.48–0.88, high: ICD score > 0.731 and low: ICD score < 0.731; Figure 2a). In addition, we further identified the distribution of ICD scores in different AJCC TNM stage subgroups (Figure 2b and Table S5). In general, the mean ICD score of the stages I and II subgroups was significantly higher than that of the stages III and IV subgroups (*p* = 0.011). Similarly, the mean ICD score was also significantly different among the different T-stage subgroups (*p* =0.003). As the different ICD subtypes exhibited different clinical outcomes, we further identified the differentially expressed genes (DEGs) in different ICD subtypes to explore the molecular mechanism regulating prognosis. The volcano plot presents ICD-related DEGs identified between the ICD-H and ICD-L subgroups (Figure 2c). In addition, the STRING database was further utilized to analyze the PPI network of the above ICD-related DEGs and found 20 hub genes including CD86, CD80, CD274, PDCD1LG2, PDCD1, HLA-DRA, MAP4K1, LCP2, VAV1, ITK, B2M, CD28, CD3E, CD3D, HLA-DRB1, CD247, CD4, CD3G, LCK, and ZAP70 (Figure 2d). These genes were all core genes in the immune response. Next, we performed GSEA of DEGs between the two ICD subtypes with the GO and KEGG databases, which identified significant differences in biological processes, cellular components, molecular functions, and signaling pathways such as the T-cell receptor signaling pathway, NK cell-mediated cytotoxicity, cell activation involved in the immune response and the immune effector process (Figure 2e, Tables S6 and S7).

### 3.3. Construction and Validation of the ICDrisk Score Model

**Figure 3:**
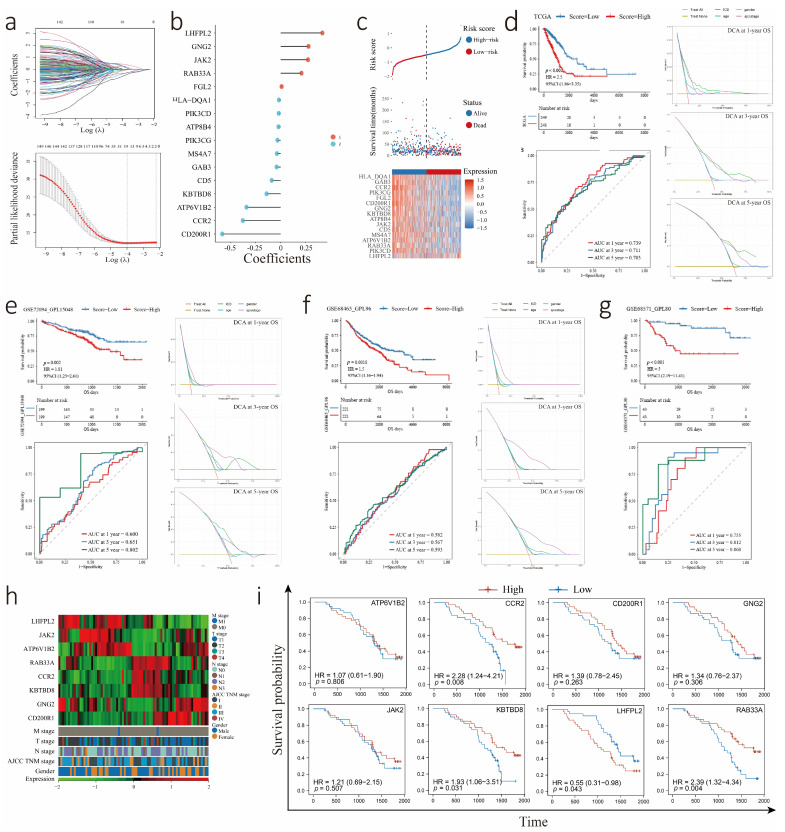
Construction and validation of the ICDrisk subtype. (**a**) Lasso Cox analysis identified 16 genes most related to OS in the TCGA-LUAD cohort. (**b**) Model coefficient of the 16 genes in ICDrisk subtype construction. (**c**) ICDrisk distribution, survival status, and heatmap of 16 differentially expressed genes among patients in TCGA-LUAD cohort. (**d**–**g**) Kaplan-Meier analysis demonstrated the prognostic value of the ICDrisk score model in TCGA-LUAD, GSE72094, GSE68465, and GSE68571 cohorts. The area under the curve (AUC) and decision curve analysis (DCA) represented the accuracy of the 16 genes model predicting the OS of patients. (**h**) The heatmap presented the qPCR expression of eight high coefficient absolute value genes and clinical features among 78 patients in Sun Yat-sen University Cancer Center (SYSUCC). (**i**) Kaplan-Meier curves showed different prognoses between high- and low-expression subtypes of eight selected genes among the SYSUCC cohort.

To further establish a prognostic model, 381 ICD-related genes were screened for subsequent analysis, and 151 potential prognostic genes were identified by univariate Cox regression analysis. Afterward, 16 genes identified from 151 genes by LASSO-Cox analysis were utilized to construct the risk score model. The prognostic value of 16 genes was evaluated in the TCGA-LUAD cohort (Figure S2). Among the 16 genes, 9 genes were negatively correlated with the OS of patients, and 5 genes were positively correlated with the OS of patients by univariate Cox analysis (Figure 3a,b). The ICDrisk score was calculated with the formula below: ICDrisk = 0.009183×FGL2 + 0.278419×GNG2 + 0.416996 × LHFPL2 − 0.207906 × RAB33A + 0.27315 × JAK2 − 0.5856 × CD200R1 − 0.37916 × CCR2 − 0.01948 × PIK3CD − 0.04153 × GAB3 − 0.0221 × ATP8B4 − 0.34285 × ATP6V1B2 − 0.09044 × CD5 − 0.03261 × PIK3CG − 0.14181 × KBTBD8 − 0.01772 × HLA-DQA1 − 0.03841 × MS4A7.

Additionally, the relationship between the ICDrisk score and survival status was investigated in our study. The results showed that patients with a low ICDrisk had a better prognosis than patients with a high ICDrisk (Figure 3c). Moreover, the prognostic value of the ICDrisk score model was demonstrated with the TCGA-LUAD cohort and further externally verified with the GEO database (GSE72094, GSE68571, and GSE68465) by utilizing Kaplan-Meier analysis, ROC curve analysis, and decision curve analysis (DCA) (Figure 3d–g). Next, we selected the eight genes with the highest absolute value for qPCR verification in SYSUCC clinical samples. The results showed that LUAD patients with low expression of CCR2, KBTBD8, and RAB33A had a poor prognosis, as did patients with high expression of LHFPL2 (Figure 3h,i).

### 3.4. Transcriptome Analysis of LUAD Cohorts Divided into Low and High ICDrisk Subtypes

**Figure 4:**
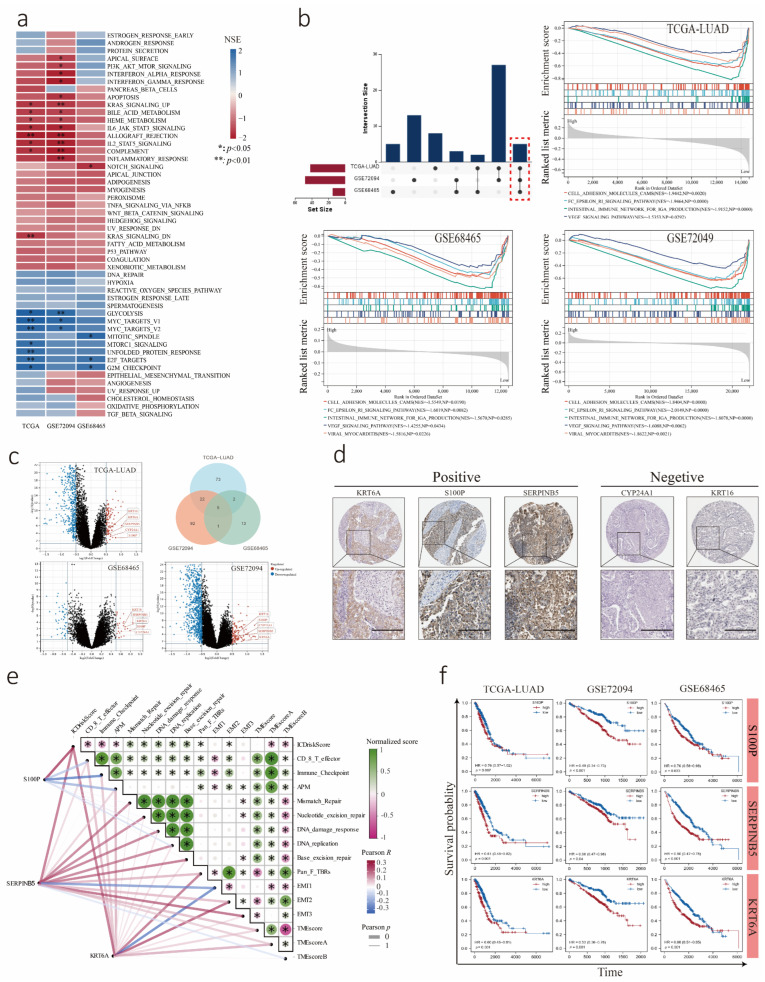
Identification of activated pathways and differentially expressed genes in the high-ICDrisk subtype of TCGA-LUAD, GSE72094, and GSE68465 cohorts. (**a**) GSEA for hallmark gene sets confirmed the activated and inhibited pathways in three databases. (**b**) GSEA for the KEGG database confirmed five common activated pathways among all three databases. The five common activated pathways are marked with a red frame. (**c**) Volcano and Venn plots represented the highly expressed genes among all three databases. (**d**) Immunohistochemical (IHC) data from the HPA database demonstrated that KRT6A, S100P, and SERPINB5 overexpressed in LUAD tumors. The black line represented 20 μm. (**e**) The correlation between KRT6A, S100P, SERPINB5, and biological functions. (**f**) Kaplan-Meier curves showed the OS with high and low expression of KRT6A, S100P, and SERPINB5 in three cohorts. *: *p* < 0.05; **: *p* < 0.01.

To explore the potential biological mechanisms between different ICDrisk subtypes, transcriptome analysis of the TCGA-LUAD, GSE72094, and GSE68465 genesets was carried out, and GSE68571 was excluded from this analysis because it covered incomplete gene numbers. GSEA confirmed the activated signaling pathways in the high ICDrisk score subtype, including MYC targets V1, MYC targets V2, and E2F targets. Some inhibited signaling pathways in the high ICDrisk score subtype were also identified, including PI3K/AKT/mTOR signaling, apoptosis, and notch signaling (Figure 4a). In addition, GSEA demonstrated that five hallmark pathways were significantly co-enriched in the low ICDrisk group among the three cohorts; these pathways included cell adhesion molecules (CAMs), the Fc epsilon RI signaling pathway, the intestinal immune network for IGA production, the VEGF signaling pathway and viral myocarditis (Figure 4b). Furthermore, we performed a correlation analysis between the ICDrisk and gene scores of important biological pathways in LUAD (Figure 4e). We found that the ICDrisk was negatively correlated with CD8 T effector, immune checkpoint, APM, TMEscoreA, and TMEscoreB, but positively correlated with mismatch repair, nucleotide excision repair, DNA damage response, DNA replication, base excision repair, and pan-fibroblast TGFβ response signature (Pan-F TBRs).

To further explore ICDrisk-related genes in LUAD, we performed differentially expressed gene analysis between the low and high ICDrisk subtypes with |log2 (fold change) | > 0.5 and *p* < 0.05 as the threshold. All five co-upregulated genes including S100P, SERPINB5, KRT16, KRT6A, and CYP24A1 were identified among the TCGA-LUAD, GSE72094, and GSE68465 cohorts (Figure 4c). To assess the expression of these five genes at the protein level, we acquired immunohistochemical (IHC) data from the HPA database. The results suggested that the protein expression of KRT6A, S100P, and SERPINB5 was positive in LUAD, but that of CY24A1 and KRT16 was negative in all samples (Figure 4d). Moreover, KRT6A, S100P, and SERPINB5 were all positively correlated with the ICDrisk score, which was closely related to the terms CD8+ T cell effector, immune checkpoint, and EMT2. In addition, S100P was negatively correlated with the terms CD8+ T cell effector and immune checkpoint, while KRT6A and SERPINB5 were positively correlated with Pan-F TBRs and negatively correlated with EMT2 (Figure 4e, Tables S8 and S9). Furthermore, we performed a survival analysis and found that LUAD patients with high KRT6A, S100P, and SERPINB5 expression had worse survival outcomes (Figure 4f).

### 3.5. Mutation Statuses of LUAD Patients with Different ICDrisk Subtypes

**Figure 5:**
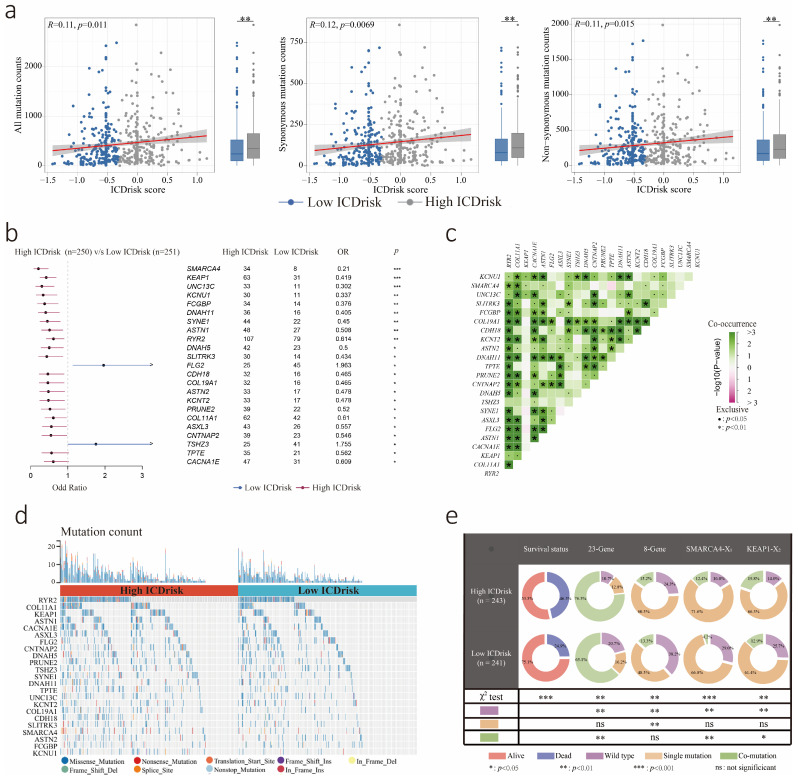
Tumor mutation status related to ICDrisk score. (**a**) Association between all mutation counts, synonymous mutation counts, non-synonymous mutation counts, and ICDrisk score, and their distribution in the low-ICD and high-ICDrisk score groups. (**b**) Forest plot of genes mutating differentially in patients of low-ICD and high-ICDrisk groups. (**c**) Interaction effect of genes mutating differentially in patients of the low-ICD and the high-ICDrisk groups. (**d**) The forms and counts of genes mutating differentially in patients of low-ICD and high-ICDrisk groups. (**e**) Constitution of wild type, a single mutation, and co-mutation among the 23 genes, 8 genes SMARCA4-X1 and KEAP1-X2. X1 represented TP53, STK11, and KRAS. X2 represented NFE2L2, TP53, STK11 and PBRM1. *: *p* < 0.05; **: *p* < 0.01; ***: *p* < 0.001; ns: not significant.

To further identify the ICD-related molecular mechanism in LUAD, the somatic mutation status of patients in the TCGA-LUAD cohort was analyzed. When compared with the low ICDrisk subtype, the high ICDrisk subtype had more somatic mutations, including synonymous and nonsynonymous mutations (all *p* < 0.05, Figure 5a). In addition, 21 mutated genes including SMARCA4, KEAP1, UNC13C, KCNU1, FCGBP, DNAH11, SYNE1, ASTN1, RYR2, DNAH5, SLITRK3, CDH18, COL19A1, ASTN2, KCNT2, PRUNE2, COL11A1, ASXL3, CNTNAP2, TPTE, and CACNA1E were observed to be more frequent in the high ICDrisk subtype of LUAD patients. However, FLG2 and TSHZ3 were more frequent in the low ICDrisk subtype of LUAD patients (all *p* < 0.05, Figure 5b and Table S10). Furthermore, we examined the mutation status of these 23 genes in LUAD patients and found many genes exhibited significant co-mutation and an increased rate of mutation in the high ICDrisk subtype (Figure 5c,d). The rate of the co-occurrence of at least two of the 23 gene mutations was 76.5% in the high ICDrisk subtype and 63.1% in the low ICDrisk subtype (*p* < 0.01, Figure 5e). To further explore the genes with potential for targeted therapy in LUAD patients, the co-mutation data of eight genes including EGFR, RET, BRAF, HER2, KRAS, ALK, ROS1, and MET were analyzed. However, there was no significant difference in the co-mutation of these eight genes between the low- and high- ICDrisk subtypes (Figure 5e). Moreover, the co-mutation of SMARCA4, TP53, STK11, and KRAS, which indicated a poor prognosis in lung cancer patients, occurred significantly more frequently in the high ICDrisk subtype (12.4%) than in the low ICDrisk subtype (4.2%) (*p* < 0.01, Figure 5e). Interestingly, the co-mutation of KEAP1, NFE2L2, TP53, STK11, and PBRM1, which exhibited a close correlation with immunotherapy, occurred significantly more frequently in the high ICDrisk subtype (19.8%) than in the low ICDrisk subtype (12.9%) (*p* < 0.05, Figure 5e).

### 3.6. Relationship between Immune Cells Infiltration and ICDrisk Subtypes

**Figure 6:**
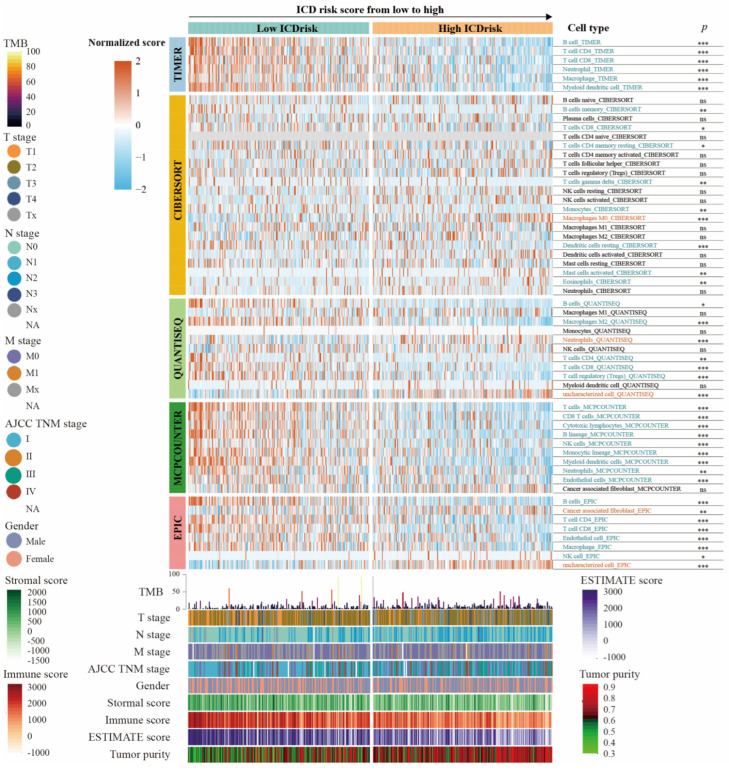
Landscape of immune and stromal cell infiltration in ICD-low and ICD-high subtypes. The heatmap presents the normalized scores of immune and stromal cell infiltrations. Blue represents the lower infiltration of cells in the ICD-high subtype and red represents the higher infiltration of cells in the ICD-high subtype. The statistical difference between ICD-low and ICD-high subtypes was defined as *p*< 0.05. The clinical features and gene mutation patterns of patients were also illustrated as an annotation. *: *p* < 0.05; **: *p* < 0.01; ***: *p* < 0.001; ns: not significant; TMB: tumor mutation burden.

Different levels of immunogenic cell death activation can lead to heterogeneity in immune cell infiltration. Hence, immune cell components and tumor purity of both ICDrisk subtypes were further identified by using the TIMER, CIBERSORT, QUANTISEQ, MCPCOUNTER, EPIC, XCELL, and ESTIMATE algorithms (Tables S11 and S12). As shown in Figure 6, there was a significant difference in immune cell composition between ICDrisk subtypes. For instance, the scores for CD8 T cells, natural killer (NK) cells, dendritic cells (DCs), and B cells were significantly higher in the low ICDrisk subtype, while that for cancer-associated fibroblasts was higher in the high ICDrisk subtype. Thus, the low ICDrisk subtype was immunologically “hot”, while the high ICDrisk subtype was immunologically “cold”.

**Figure 7:**
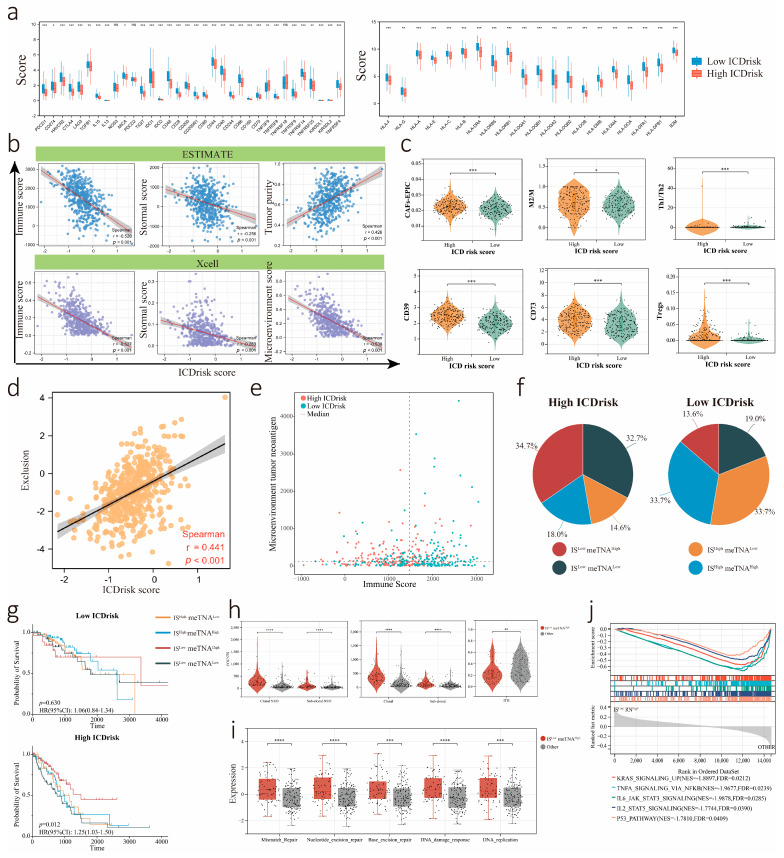
Analysis for correlation between ICDrisk subtypes and tumor microenvironment. (**a**) Analysis for expression of HLA family genes and immune checkpoints between ICDrisk subtypes. (**b**) Association between immune score, stromal score, tumor purity, microenvironment score, and ICDrisk score by using the TIMER and XCELL algorithms. (**c**) The count and proportion of clonal and sub-clonal between low and high ICDrisk score subgroups. (**d**) The distribution of microenvironment tumor neoantigen (meTNA) and immune score (IS) between low and high ICDrisk score subgroups. The dotted line represented the median of meTNA and IS. (**e**) The pie represented the proportion of IS^low^ meTNA^high^, IS^low^ meTNA^low^, IS^high^ meTNA^low^, and IS^high^ meTNA^high^. (**f**) The violin plot represented the distribution of CAFs-EPIC, M2M, MDSC, and Th1/Th2 between low and high ICDrisk score subgroups. (**g**) The survival curves represented the OS of IS^low^ meTNA^high^, IS^low^ meTNA^low^, IS^high^ meTNA^low^, and IS^high^ meTNA^high^ between low and high ICDrisk score subgroups. (**h**,**i**) The violin plot and box plot represented the distribution of variates between IS^low^ meTNA^high^ and other subgroups. (**j**) GSEA confirmed the suppressive pathways in IS^low^ meTNA^high^ subgroup. *: *p* < 0.05; **: *p* < 0.01; ***: *p* < 0.001; ****: *p* < 0.0001; ns: not significant.

Given the importance of immune checkpoints (ICPs) and the HLA family in anticancer immunity, we further investigated the gene expression of 34 immune checkpoint molecules and 20 HLA family genes in both ICDrisk subtypes. Patients with a high ICDrisk in the TCGA-LUAD cohort had significantly higher expression of 31 immune checkpoint molecules and all HLA family genes, while only TNFSF9 was more highly expressed in the high ICDrisk subtype (Figure 7a). In addition, increases in the immune and stromal scores were associated with a decrease in the ICDrisk, while an increase in tumor purity was associated with an increase in the ICDrisk (Figure 6 and Figure 7b).

### 3.7. Association between the LUAD Immune Signature and ICDrisk Score

TIDE scoring indicated that high ICDrisk tumors had a microenvironment characterized by tumor immune exclusion (Figure 7d). Patients with a high ICDrisk exhibited multifactor immunosuppression, and there were high proportions of CD39+ cells, CD73+ cells, Treg cells, Th1/Th2 cells, tumor-associated fibroblasts (CAFs), and M2 macrophages (Figure 7c). Tumor-infiltrating lymphocytes and tumor neoantigens (TNAs) are two crucial factors affecting immunogenic cell death. Immune suppression and exclusion activity become increasingly significant with the malignant progression of tumors. Therefore, the ICDrisk of LUAD patients was further analyzed by these two dimensions. Due to the differences in tumor purity among the studied tumor tissues, to better measure the relationship between these dimensions in the tumor microenvironment (TME), we used the tumor purity value obtained by the ESTIMATE algorithm to correct clonal neoantigen counts (TME-neoantigen (meTNA) = clonal neoantigen counts/tumor purity). Patients were divided into four subgroups based on the median value of the immune score (IS) and meTNA, namely, the IS^low^meTNA^high^, IS^low^meTNA^low^, IS^high^meTNA^low^, and IS^high^meTNA^high^ subgroups (Figure 7e). In addition, these four subgroups accounted for different proportions in the low and high ICDrisk subgroups (Figure 7f). Moreover, the survival of the IS^low^meTNA^high^ subtype was significantly better than that of the other three groups within the high ICDrisk subtype (Figure 7g). Our results demonstrated that intratumor heterogeneity (ITH) was lower in the IS^low^meTNA^high^ subtype, while the numbers of clonal neoantigens and subclonal neoantigens were higher in the IS^low^meTNA^high^ subtype (Figure 7h). Additionally, biological pathway analysis of the subgroups showed that mismatch repair, nucleotide excision repair, base excision repair, DNA damage response, and DNA replication were more active in the IS^low^meTNA^high^ subtype. GSEA was further performed to compare the IS^low^meTNA^high^ subtype with the other subgroups within the high ICDrisk group, which showed that KRAS signaling upregulation, TNFA signaling via NFκB, IL6-JAK-STAT3 signaling, IL3-JAK-STAT3 signaling, and the P53 pathway were less active in the IS^low^meTNA^high^ subtype (Figure 7i,j).

### 3.8. Predictive Value of the ICDrisk for Response to Immunotherapy and Prognosis of LUAD Patients

**Figure 8:**
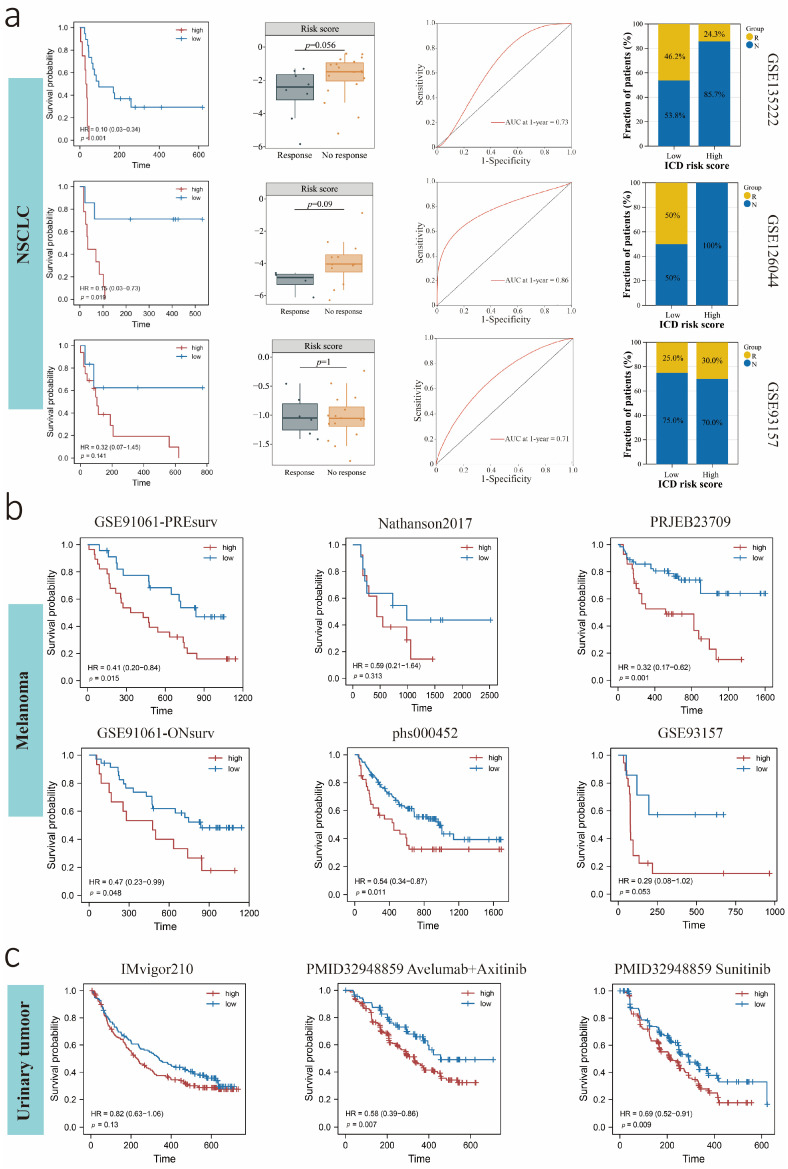
Prediction of survival and response to immunotherapy between high and low ICDrisk subgroups among non-small cell lung cancer (NSCLC) (**a**), melanoma (**b**), and urinary tumor (**c**). The survival curves represented the OS of low and high ICDrisk subgroups among patients treated with immunotherapy. The box plot represented the distribution of ICDrisk scores among patients with or without response to immunotherapy. The area under the curve (AUC) represented the accuracy of predicting the response to immunotherapy. The bar chart represented the percentage of patients with or without response to immunotherapy between low and high ICDrisk subgroups.

We further identified a role for ICDrisk in predicting response to immunotherapy and prognosis among LUAD patients. The results showed that the subgroup responding to immunotherapy in LUAD had a lower ICDrisk, and the low ICDrisk subtype had better survival outcomes. Moreover, ROC curves indicated excellent performance for the ICDrisk in predicting the 1-year overall survival (OS) of LUAD patients, with an AUC greater than 0.7 in each dataset (Figure 8a). To further explore the predictive efficiency of the ICDrisk in other cancers, we analyzed melanoma and urinary tumor data. The results showed that the ICDrisk had a consistent predictive value in melanoma (Figure 8b) and urinary tumors (Figure 8c), as in LUAD.

## 4. Discussion

A comprehensive analysis of the intrinsic immunogenic cancer cell death property of LUAD will provide new information for tumor vaccines and ICD inducers and potential markers for the assessment of prognosis and the efficacy of immune checkpoint inhibitors. In our study, the ICDrisk subtypes were identified based on the TCGA-LUAD cohort, and multiple external validation analyses were performed to predict prognosis in LUAD cohorts. Our findings clarify the relationship between clinicopathological features and ICD subtypes. The two ICD subtypes had distinct clinical prognoses, immune infiltration microenvironments, somatic mutation statuses, and activated molecular functions. Patients with a low ICDrisk were associated with more active signaling pathways related to the immune response, higher immunogenicity, fewer somatic mutations, higher immune and stromal cell infiltration and lower tumor purity than patients with a high ICDrisk. Furthermore, as far as we know, this is the first subgroup analysis based on two key elements of immunogenic tumor cell death: immune infiltration and tumor neoantigens. Interestingly, we found that tumors with both low immune infiltration and a high endogenous neoantigen load had a better prognosis. Finally, the ICDrisk could be a potential predictive biomarker for prognosis in LUAD and immunotherapeutic response in Pan-cancer.

Previous studies have demonstrated that ICD plays a key role in driving the adaptive immune response and establishing long-term immune memory [33]. Previous studies on ICD have mostly focused on factors with the potential to improve tumor immunogenicity and antigen presentation, including cellular stressors; conventional chemotherapeutic drugs, such as cyclophosphamide, oxaliplatin, and docetaxel [34,35]; tyrosine kinase inhibitors, such as crizotinib [36]; the epidermal growth factor receptor (EGFR) specific monoclonal antibody cetuximab [37]; oncolytic virotherapy [38,39,40]; epigenetic modifiers; and numerous physical interventions [41,42,43,44]. At present, there is a lack of omni-directional, large-scale, and multiomic analyses of ICD characteristics in lung adenocarcinoma patients. Therefore, it is necessary to carry out ICD property analysis in both pretreatment and intrinsic tumors for a more comprehensive understanding of the effects of intrinsic immunogenic tumor death cell characteristics on prognosis, gene mutation, and tumor microenvironment, thus providing guidance for the development of prophylactic tumor vaccines and ICD inducers.

We used the WGCNA method to screen gene sets that were highly synergistic with intrinsic ICD properties. Enrichment analysis showed that these genes were highly enriched in immune response-related functions, which might play key roles in improving intrinsic immunogenicity and activating cytotoxic T lymphocytes, consistent with the mechanism of ICD. Next, using the ICD score obtained from ICD-related genes, we hypothesized that ICD-H was associated with better survival and an earlier stage, implying that this score could predict prognosis in LUAD, which also illustrated that there were different subtypes defined by ICD properties in large-scale LUAD patient cohorts. To accurately predict the prognosis of patients and further understand the subtype of ICD, we performed survival and regression analyses based on ICD-related genes. We identified ICDrisk subtypes, and the external validation results showed that the ICDrisk could be a reliable prognostic indicator for LUAD patients.

The activation of some pathways was confirmed in the high ICDrisk subtype, including MYC targets, E2F targets, and the G2M checkpoint [45,46,47]. The above-activated pathways further explained the worse survival in the high ICDrisk subtype. Moreover, the down-regulation of cell adhesion molecules (CAMs) and the Fc epsilon RI signaling pathway were consistently confirmed in the high ICDrisk subtype in the three cohorts. Alterations in these pathways were proven to enhance cancer progression and metastasis, inhibit the adaptive immune system, and contribute to immune evasion [48,49]. In this study, we wanted to collect as many immune cell signatures as possible to explore biological functions, similar to the immune cell signature collection work of Sanjeev Mariathasan et al. [50]. Therefore, the cell signatures of 15 identified biological functions were collected. The above results indicated that tumors with a high ICDrisk might be associated with malignant progression and immunosuppression. Furthermore, high expression levels of KRT6A, S100P, and SERPINB5 were consistently observed in patients with a high ICDrisk and associated with a poor prognosis, which might play critical roles in the ICD process. In previous studies, these gene families were defined as a panel affecting innate immunity and cell differentiation in different diseases [51,52]. However, the functions of these genes in lung cancer still need to be further explored.

Immunogenic cell death involves a multi-parameter interaction between dying cells and the immune system [33]. Simply, there are three key elements involved in the activation of ICD: an adjuvant, antigenicity, and microenvironmental factors, which are often produced by different cells in the tumor microenvironment, thus forming a complex molecular mechanism. Adjuvants usually refer to DAMPs, cytokines, and chemokines, which are mainly released by dying cancer cells first and are necessary for the recruitment and maturation of antigen-presenting cells (APCs), which further drives a cytotoxic T-lymphocyte (CTL)-dependent immune response [53,54,55]. In addition, DCs are key mediators in many antitumor treatments, including immunotherapy, chemotherapy, and radiotherapy, that act by regulating ICD [56]. In this study, patients with a low ICDrisk exhibited high DCs infiltration and high expression of HLA family genes, which also suggested that with the occurrence of ICD, not only were antigen-presenting cells recruited but also the subtype had a stronger antigen-presenting function. Microenvironmental factors are regulated by immune cells and stromal cells. At present, the direction of ICD inducer-based therapy is to stimulate tumor immunogenicity and transform “cold” tumors lacking immune infiltration into “hot” tumors rich in immune infiltration. Our study found that with an increase in the ICDrisk, tumors gradually transformed from a “hot” tumor to a “cold” tumor; meanwhile, the purity of the tumor gradually increased until ultimately becoming an immune-desert phenotype. In addition, some studies have noted that ICD inducers can enhance the efficacy of immune checkpoint inhibitors on the premise of maintaining the activity of tumor-infiltrating lymphocytes or normal immunostimulatory signaling [57,58]. In our study, patients with a high ICDrisk had a higher level of immune checkpoint gene expression, and we confirmed that ICDrisk subtypes could indicate efficacy through multiple independent immunotherapy cohorts of NSCLC, melanoma, and urinary tumors. These findings verified our hypothesis that ICDrisk subtypes can be used clinically to effectively predict the efficacy of immunotherapy.

In the process of tumor development, tumor neoantigens (TNAs) appear with somatic mutations, and they have both high tumor specificity and high immunogenicity [59]. With the improvement of next-generation sequencing technology, people can further evaluate the level of neoantigen immunogenicity [60]. Under continuous immune pressure, the mutational load and TNA landscape change with tumor progression and immune infiltration heterogeneity, and they are affected by increased genomic instability and intervention with different treatments, resulting in tumor microenvironments with differences in antigenicity [61,62]. TNAs also show intratumoral heterogeneity, as some are found in all tumor cells (clonal neoantigens) and some are found only in some cells (subclonal neoantigens). Many studies have noted that clonal neoantigens have sufficient antigenicity to drive an immune response, so the tumor microenvironmental neoantigens (meTNAs) defined in this study not only include clonal neoantigens as the main research object but can also eliminate differences caused by tumor purity to some extent. In addition, effective TNAs reach the threshold of T-cell recognition, thus breaking immune tolerance and successfully inducing an antitumor immune response [63,64]. Many studies have demonstrated that higher neoantigen load correlated with both higher cytotoxin release activity of T lymphocytes and better prognosis. However, owing to the influence of immunosuppressive cells and immunosuppressive factors in the immune microenvironment, neoantigens cannot be effectively recognized by autologous T lymphocytes [65,66]. Therefore, we hypothesized that with the change in the relationship between the level of immune infiltration in the tumor microenvironment and the number of TNAs, the efficiency of T lymphocytes in recognizing TNAs also changes dynamically. Subgroup analysis suggested that the IS^low^meTNA^high^ subtype had a better prognosis than the high ICDrisk subtype. These findings not only more accurately identified the subcluster with the best immunogenicity but also suggested that high antigenicity and low levels of immunosuppressive cell infiltration in the microenvironment were more important. In addition, our further analysis showed that the IS^low^meTNA^high^ subtype showed lower tumor heterogeneity, and biological function analysis suggested that this subgroup had a stronger DNA damage repair ability for maintaining genomic stability and suppressed tumor progression-related pathways. Further study of these phenomena will help achieve an accurate diagnosis and effective treatment, as well as contribute to the development of ICD inducers and ICD-related tumor vaccines.

Despite some limitations, TMB is still considered a useful biomarker of immunotherapy response, with higher TMB suggesting greater immunotherapy benefit [67]. In our study, it was demonstrated that patients with high ICDrisk had high TMB. However, as discussed above, the high ICDrisk subtype showed lower immune activity, suggesting that high TMB did not necessarily predict high immunogenicity. Furthermore, the most common mutations in the high ICDrisk group were in 21 genes, which could explain the high TMB. Interestingly, the frequency of co-mutations in these genes was high in the high ICDrisk group. Among these genes, SMARC4 and KEAP1 were more frequently mutated in the high ICDrisk group. As previous studies have reported, the co-mutations of SMARCA4, TP53, STK11, and KRAS occurred more frequently in SMARCA4-mutant lung cancer, indicating worse survival and increased sensitivity to immunotherapy [68]. A previous study revealed that the co-mutation of KEAP1 and TP53 influenced the prognosis in LUAD by affecting the immune microenvironment composition, which played a crucial role in immunotherapy [69,70]. LUAD with co-mutation of KEAP1 and NFE2L2 was also proven to be relatively responsive to immunotherapy [71]. However, cases with co-mutation of KEAP1, STK11, and PBRM1 were proven to be unresponsive to ICI treatment in LUAD [72]. In our study, the rates of both single mutations and co-mutations of SMARCA4, TP53, STK11, KRAS KEAP1, NFE2L2, STK11, and PBRM1 were higher in the high ICDrisk group, which had a high TMB and low immune checkpoint molecule expression.

This study still had some limitations. First, we used the transcriptome expression level of the ICD metagene signature to represent the intrinsic ICD status. Although we used a classic gene signature algorithm to elucidate the tumor landscape, due to technical and methodological limitations, the actual ICD status was not fully recognized. Second, although the high ICDrisk subtype was found to indicate a poor prognosis in many LUAD cohorts, the biological or medical mechanism underlying this is still not very clear. Additional experimental studies on the mechanism underlying immunogenic cell death characteristics are needed to provide more important information to improve the understanding of the functional roles of these characteristics in LUAD. Third, the ICD-related genes screened in this study need to be subjected to a series of in vitro and in vivo studies to explore their functions. Finally, we are also aware that this study lacks a large-scale and independent sequencing database, and the reliability and strength of the conclusions are limited. Due to the limited public data available for the NSCLC immunotherapy cohort, we used a small number of NSCLC cases to verify the effectiveness of the ICDrisk in the prediction of immunotherapy response, so we are collecting relevant cases from an affiliated hospital to complete the validation of the LUAD results, which is listed as our next priority.

## 5. Conclusions

In summary, we effectively identified ICDrisk subtypes to predict the OS of LUAD patients, which were externally and extensively validated. Functionally, the ICDrisk score was correlated with the immune response in pan-cancer patients. Favorable performance in validation datasets suggested the robust and extensive potential for utilization.

## Data Availability

The data that support the findings of this study are available in The Cancer Genome Atlas (TCGA) and Gene Expression Omnibus (GEO) database, reference number are described in the Materials and Methods.

## Supplementary Materials

The following supporting information can be downloaded at: 549 https://www.mdpi.com/article/10.3390/biology12060808/s1, Supplementary Figure S1. Differential expression analysis of 34 ICD-related parameters in The Cancer Genome Atlas–Lung adenocarcinoma cohort. (a) Variation analysis of 34 ICD-related parameters expression in normal tissue and tumor. (b) Variation analysis of 34 ICD-related parameters expression in different pathological stages. (c) The left panel presents the relationship between the soft-threshold and scale-free R2. The right panel presents the relationship between the soft-threshold and mean connectivity. (d) Verification of the scale-free network; Supplementary Figure S2. Kaplan–Meier analyses for the prognostic prediction of 16 ICD-related genes of risk score model in The Cancer Genome Atlas–Lung adenocarcinoma cohort, respectively; Table S1. Summary table of public datasets used in the present study; Table S2. Oligonucleotide sequences used in the present study; Table S3. ICD level of patients in TCGA-LUAD cohort; Table S4. KEGG enrichment analysis for 381 ICD-related genes; Table S5. Clinicopathological data and ICDrisk score of TCGA-LUAD samples; Table S6. GO and KEGG enrichment analysis for ICD-related genes; Table S7. GSEA data for high- and low-ICD levels of TCGA-LUAD samples; Table S8. Correlation analysis data between biological functions and high-ICDrisk-related genes; Table S9. Co-mutation data for high- and low-ICDrisk samples; Table S10. Summary of the gene sets and markers of indicated biological processes in our study.; Table S11. Immune infiltration data for TCGA-LUAD samples; Table S12. Estimate data for TCGA-LUAD samples.
