# Supplementary figures and images for: Intrinsic Immunogenic Tumor Cell Death Subtypes Delineate Prognosis and Responsiveness to Immunotherapy in Lung Adenocarcinoma
Source: Biology (Basel). 2023 Jun 2;12(6):808. doi: 10.3390/biology12060808 (PMC10295033; doi:10.3390/biology12060808)

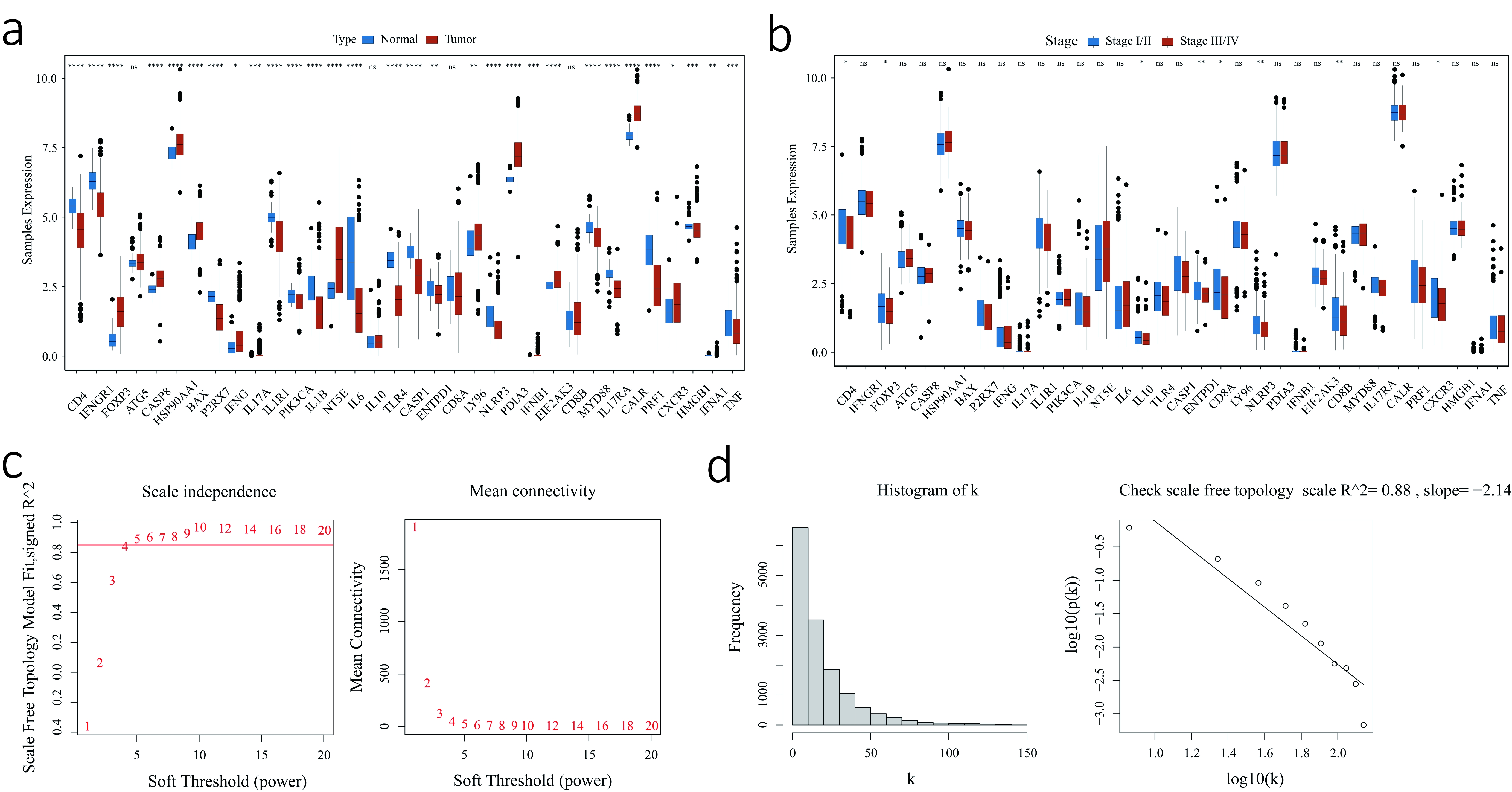

Supplement: Supplementary file 1 [file biology-12-00808-s001.zip › Supplementary Figure S1.tif]

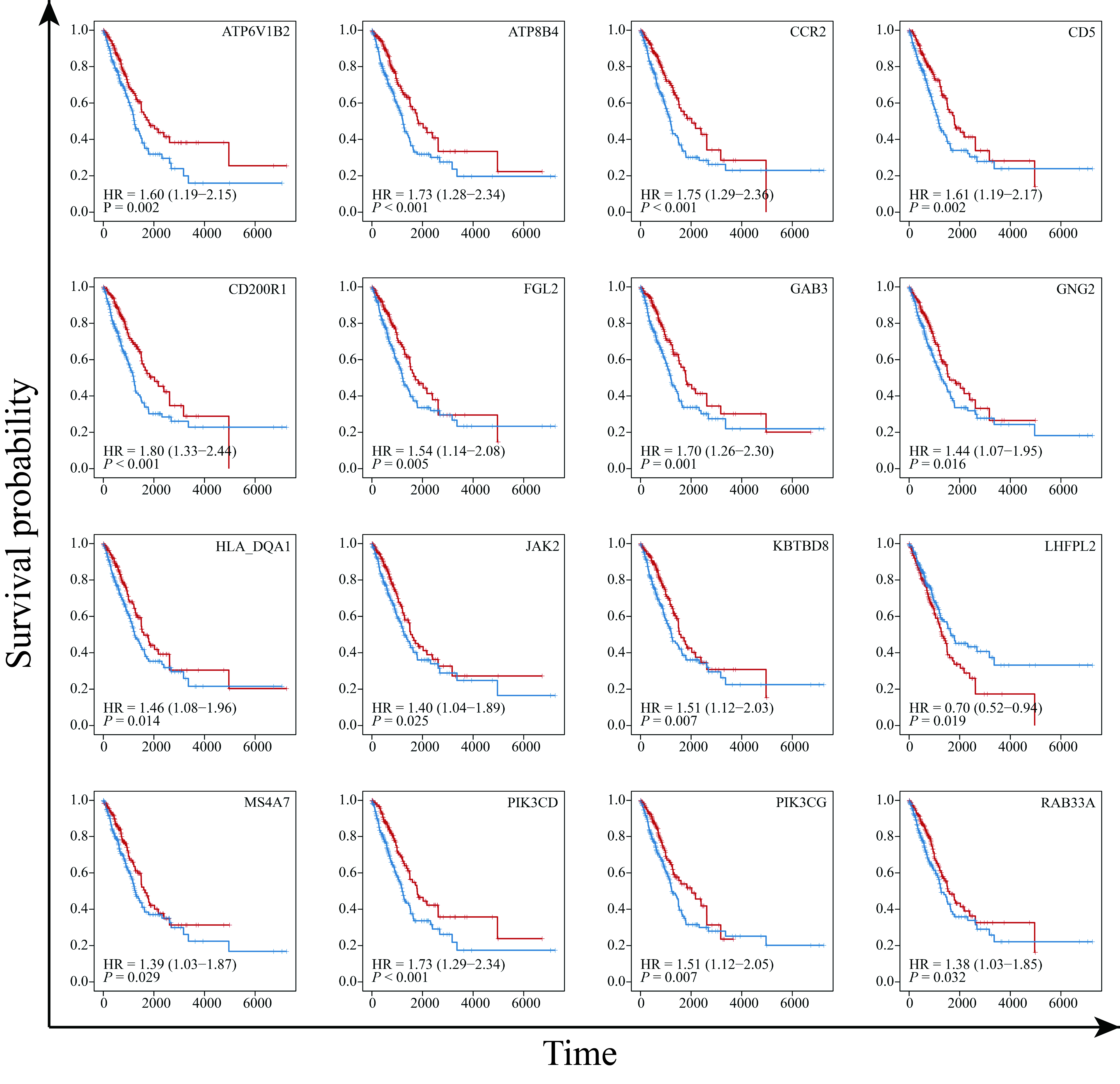

Supplement: Supplementary file 1 [file biology-12-00808-s001.zip › supplementary Figure S2.tif]
